# Gene Expression, Single Nucleotide Variant and Fusion Transcript Discovery in Archival Material from Breast Tumors

**DOI:** 10.1371/journal.pone.0081925

**Published:** 2013-11-22

**Authors:** Nadine Norton, Zhifu Sun, Yan W. Asmann, Daniel J. Serie, Brian M. Necela, Aditya Bhagwate, Jin Jen, Bruce W. Eckloff, Krishna R. Kalari, Kevin J. Thompson, Jennifer M. Carr, Jennifer M. Kachergus, Xochiquetzal J. Geiger, Edith A. Perez, E. Aubrey Thompson

**Affiliations:** 1 Department of Cancer Biology, Mayo Clinic, Jacksonville, Florida, United States of America; 2 Department of Health Sciences Research, Mayo Clinic, Rochester, Minnesota, United States of America; 3 Department of Health Sciences Research, Mayo Clinic, Jacksonville, Florida, United States of America; 4 Medical Genome Facility, Mayo Clinic, Rochester, Minnesota, United States of America; 5 Department of Laboratory Medicine and Pathology, Mayo Clinic, Jacksonville, Florida, United States of America; 6 Department of Medicine, Mayo Clinic, Jacksonville, Florida, United States of America; University of Connecticut Health Center, United States of America

## Abstract

Advantages of RNA-Seq over array based platforms are quantitative gene expression and discovery of expressed single nucleotide variants (eSNVs) and fusion transcripts from a single platform, but the sensitivity for each of these characteristics is unknown. We measured gene expression in a set of manually degraded RNAs, nine pairs of matched fresh-frozen, and FFPE RNA isolated from breast tumor with the hybridization based, NanoString nCounter (226 gene panel) and with whole transcriptome RNA-Seq using RiboZeroGold ScriptSeq V2 library preparation kits. We performed correlation analyses of gene expression between samples and across platforms. We then specifically assessed whole transcriptome expression of lincRNA and discovery of eSNVs and fusion transcripts in the FFPE RNA-Seq data. For gene expression in the manually degraded samples, we observed Pearson correlations of >0.94 and >0.80 with NanoString and ScriptSeq protocols, respectively. Gene expression data for matched fresh-frozen and FFPE samples yielded mean Pearson correlations of 0.874 and 0.783 for NanoString (226 genes) and ScriptSeq whole transcriptome protocols respectively, p<2x10^-16^. Specifically for lincRNAs, we observed superb Pearson correlation (0.988) between matched fresh-frozen and FFPE pairs. FFPE samples across NanoString and RNA-Seq platforms gave a mean Pearson correlation of 0.838. In FFPE libraries, we detected 53.4% of high confidence SNVs and 24% of high confidence fusion transcripts. Sensitivity of fusion transcript detection was not overcome by an increase in depth of sequencing up to 3-fold (increase from ~56 to ~159 million reads). Both NanoString and ScriptSeq RNA-Seq technologies yield reliable gene expression data for degraded and FFPE material. The high degree of correlation between NanoString and RNA-Seq platforms suggests discovery based whole transcriptome studies from FFPE material will produce reliable expression data. The RiboZeroGold ScriptSeq protocol performed particularly well for lincRNA expression from FFPE libraries, but detection of eSNV and fusion transcripts was less sensitive.

## Introduction

The wealth of clinical data such as patient outcome and disease-free survival from large clinical trials is an invaluable tool in the successful application of genomic technologies. More than one billion samples are preserved with formalin fixed paraffin embedding (FFPE) in hospitals and tissues banks across the world [1]. Unfortunately, this method of preservation causes chemical modification and degradation of RNA, compromising the use of genomic technologies such as whole transcriptome sequencing [2]. 

Formalin induces chemical modification by crosslinking between nucleic acids and proteins, limiting the reverse transcription of mRNA into cDNA, a key step in the process of library preparation for transcriptome sequencing. Further challenges originate from the enrichment procedure of mRNA from the total RNA fraction and subsequent 3’ amplification bias when starting material is fragmented. There are three potential solutions.

First, formalin induced chemical modifications are partially reversible [3], a process now included in many commercially available nucleic acid extraction kits for FFPE material, such as the one used in this study. A second alternative for accurate gene expression quantitation from FFPE or fragmented material is to avoid cDNA synthesis and subsequent amplification steps altogether, a method employed by the NanoString nCounter system, based on direct measurement of transcript abundance using multiplexed color-coded probe pairs (up to 800 per assay). There are now a number of publications demonstrating the accuracy and precision of the NanoString platform with FFPE material [4–6] and its superiority to real-time quantitative PCR with RNA extracted from FFPE material [6]. The NanoString platform relies on prior selection of gene probes, making it the ideal platform for analytical validation of a subset of several hundred genes. The third alternative is to use a whole transcriptome sequencing protocol which avoids 3’ amplification bias in fragmented RNA. This can be achieved by mRNA selection using depletion of rRNA [7–11] and cDNA synthesis steps with random hexamers. There are now several commercially available kits that employ these methods, but as far as we know, their capability with FFPE material is unquantified.

The goal of this study was to first assess gene expression using degraded material with the hybridization-based, NanoString nCounter platform (thus avoiding 3’ amplification bias) and RiboZeroGold / ScriptSeq library preparation (Epicentre) (a rRNA depletion method of mRNA enrichment, designed to avoid 3’ amplification bias of degraded material), followed by Illumina sequencing. Our experimental design compared manually degraded RNA against the same sample in its original undegraded condition and matched fresh-frozen / FFPE pairs. A high degree of correlation of gene expression between degraded and undegraded sample sets with these genomic applications would indicate the potential of archival material for discovery of gene expression patterns relevant to breast cancer. In addition, use of whole transcriptome RNA-Seq is often described as advantageous over hybridization-based platforms such as the NanoString nCounter or microarrays because next generation sequencing based technologies allow not only quantitation of gene expression, but also simultaneous discovery of genomic features such as single nucleotide variants (SNVs) and fusion transcripts. Hence, we also assessed the capability of the RiboZeroGold / ScriptSeq protocol for discovery of expressed SNVs and fusion transcripts within FFPE material. 

## Materials and Methods

### Sample sets

Gene expression was assessed in two sample sets. First, we took high quality RNA from a breast cancer cell line (MDA-MBA-436) and the Universal Human Reference RNA (Stratagene, La Holla, CA) and manually degraded aliquots of each by heat and physical shearing. This resulted in a total of 10 RNA samples with RNA integrity (RIN) values ranging 1.2-10, described in Table 1. Second, we used a set of RNA samples from nine matched pairs of fresh-frozen and FFPE breast tumor (Table 2). Fresh-frozen tumor material was collected at the time of surgical resection, snap-frozen in liquid nitrogen, and stored at -80°C until RNA extraction. Clinical data for these samples is shown in Table S1.

**Table 1:** Manually degraded sample characteristics.

**Sample set A**	**Degradation status**	**RIN score**
MDA-MBA-436	Undegraded	10
MDA-MBA-436	Medium (by heat)	6.8
MDA-MBA-436	Medium (by shearing)	6.1
MDA-MBA-436	High (by heat)	2.2
MDA-MBA-436	High (by shearing)	1.2
UHRR	Undegraded	8.1
UHRR	Medium (by heat)	4.7
UHRR	Medium (by shearing)	5.2
UHRR	High (by heat)	1.8
UHRR	High (by shearing	1.7

**Table 2:** Matched FFPE and fresh-frozen sample characteristics.

**Sample set B**	**FFPE sample age at time of RNA extraction (years)**	**RIN score FFPE**	**RIN score fresh-frozen**
BRB123	1.83	2.3	8.1
BRB144	1.50	2.1	7.7
BRB147	1.42	2.2	7.0
BRB157	1.42	2.1	7.3
BRB212	1.00	2.0	7.7
BRB215	1.00	2.2	8.4
BRB248	0.83	2.2	7.8
BRB277	0.42	1.8	6.9
MCJBCR-028	4.33	2.3	8.9

FastQ files are available at GEO (accession number GSE51124).

### Ethics Statement

All breast tumor samples were collected between 2008 and 2012 according to a protocol that was approved by the Mayo Clinic Institutional Review Board with written informed consent, and were de-identified for this work. 

### RNA preparation

RNA extraction from the breast cancer cell line, MDA-MBA-436 was performed with the miRNeasy Kit (Qiagen, Inc, CA). The UHRR RNA was purchased directly from Stratagene. Both samples were treated with DNase (Qiagen, Inc, CA). Each sample was divided into aliquots, with one aliquot left undegraded, and each remaining aliquot degraded under one of the following conditions: 1) 37°C for 48 hours; 2) 65°C overnight; 3) shearing by Covaris^TM^ (Woburn, MA) under target base pair peak conditions for 1500bp (10 second then 15 second bursts) and 4) shearing by Covaris^TM^ under target base pair peak conditions for 1500, 800 and 200bp (10 second then 15 second bursts). The 1500, 800 and 200bp sheared aliquots were then combined to provide a range of different fragment sizes. Agilent Bioanalyzer profiles are shown in Figure S1A.

RNA extraction from fresh-frozen material was performed with the Qiagen miRNeasy kit, including on-column treatment with DNase. RNA extraction from FFPE material was performed on 6x10µm sections with the Qiagen AllPrep FFPE kit, including on-column treatment with DNase. All RNA samples were assessed for quality using the RNA 6000 Nano assay on the 2100 Bioanalyzer (Agilent Tehcnologies, Inc) and for quantity by Nanodrop 2000c (Thermo Scientific). Agilent Bioanalyzer profiles are shown in Figure S1B.

### NanoString gene expression quantification

Gene expression on the NanoString platform was assessed with the NanoString Cancer Gene expression panel of 226 genes known to be differentially expressed in cancer. 200ng of each total RNA sample was prepared as per the manufacturer’s instructions. Gene expression was quantified on the NanoString nCounter^TM^ and raw counts were generated with nSolver^TM^. Raw counts per gene were extracted using the nSolver software, (Nanostring). Raw gene counts were read into R (2.15.0) and each dataset (Fresh-frozen RNA and FFPE RNA) was separately quantile normalized via the Bioconductor (2.11) package “aroma.light”. Log_2_-transformed distributions, before and after quantile normalization, are shown in Figure S2. 

### RNA-Seq library preparation

rRNA depletion was performed on 200-400ng of each total RNA sample with the RiboZeroGold (Illumina, San Diego, CA) as per the manufacturer’s instructions. The entire rRNA-depleted fraction (ranging 4-22ng) was used as input for library preparation using the ScriptSeq V2 library preparation kit (Illumina, San Diego, CA) as per the manufacturer’s instructions with one exception: For the degraded and FFPE samples, the cDNA synthesis step at 37°C was increased from 10 minutes to 90 minutes. Each library was indexed with Illumina compatible barcodes to allow multiplexing.

In addition to ScriptSeq libraries, for the RNA isolated from fresh-frozen tumor, we also made TruSeq V2 libraries as per the manufacturer’s instructions (Illumina, Inc, San Diego, CA).

All libraries were validated and quantified with the Bioanalyzer DNA 1000 assay (Agilent Technologies, Inc, CA) and further quantified with the Qubit DNA Broad Range assay (Life Technologies, Carlsbad, CA). 

### RNA Sequencing

10µL of each library were diluted to a concentration of 10nM. Equal volumes of each 10nM library were then pooled for subsequent 51 base paired-end sequencing on an Illumina HiSeq 2000 (Illumina). For the manually degraded ScriptSeq V2 and the fresh-frozen tumor TruSeq V2 libraries, sequencing was performed with two samples per lane. For the ScriptSeq fresh-frozen and FFPE libraries, sequencing was performed with three samples per lane. 

Sequence alignment and gene level expression quantification: To account for higher coverage in TruSeq samples (only two per lane, verses three per lane in the ScriptSeq), and to make fair comparisons among the samples with different sequence depths, we randomly selected ~56 million reads (the smallest number of reads observed in a single sample) from each other sample. Sequence alignment and quantification of gene and exon level expression was carried out using our internally developed RNA-Seq analytical pipeline, MAPRSeq 1.2. Briefly, the paired end reads were aligned to the human genome build 37.1 using TopHat (2.0.6). HTSeq (0.5.3p9) was used to perform gene counting while BEDTools (2.16.2) was used to count the reads mapping to individual exons according to RefSeq gene annotations (Feb 2009, GRCh37/hg19) with 23,498 genes.

### Expressed single nucleotide variant (eSNV) calls

Reads not uniquely mapped were discarded. Duplicate reads were marked with Picard and removed prior to SNV calling with GATK. eSNV inclusion criteria were a quality score ≥30, total read depth ≥10 and a read depth of ≥2 for the alternate allele.

### Fusion transcript detection

Fusion transcripts were detected as previously described in Asmann et al, [12]. Parameters used to define a fusion transcript were at least two unique split reads within the dataset and at least three encompassing reads.

### Statistical analysis

Raw gene counts were read into R (2.15.0) and each RNA sample set (manually degraded, undegraded, fresh-frozen and FFPE) for each platform (Nanostring and Illumina RNASeq), and each RNASeq protocol (ScriptSeq and TruSeq), was separately quantile normalized via the Bioconductor (2.11) package “aroma.light”. Log_2_ transformed distributions, before and after quantile normalization, are shown in Figure S2. Correlation between paired samples on the same platform was assessed by Spearman’s and Pearson’s correlation coefficients. The power to detect differences given various states of degradation was determined by comparing the log_2_ fold change between undegraded MDA-MB-436 and UHRR versus the log_2_ fold change in the degraded form on the same platform. These fold changes were also compared via Spearman’s and Pearson’s correlation coefficients.

Cross-platform agreement was assessed using both Spearman’s and Pearson’s correlation coefficients on a per-sample basis. 

All correlation p-values were calculated via Fisher’s z-transformation.

## Results

### RNA sample yield and quality

RNA extraction from 6x10µM FFPE sections yielded a mean of 7.99µg per sample (ranging 3.96-15.03µg). RNA extraction from 10x10uM sections of fresh-frozen tumor yielded a mean of 7.75ug per sample (ranging 1.8-22.1ug). RIN scores and FFPE sample age at the time of RNA extraction are shown in Table 2. Agilent Bioanalyzer profiles are shown for each sample in Figure S1.

### Cancer gene expression panel: NanoString nCounter^TM^

**Figure 1:**
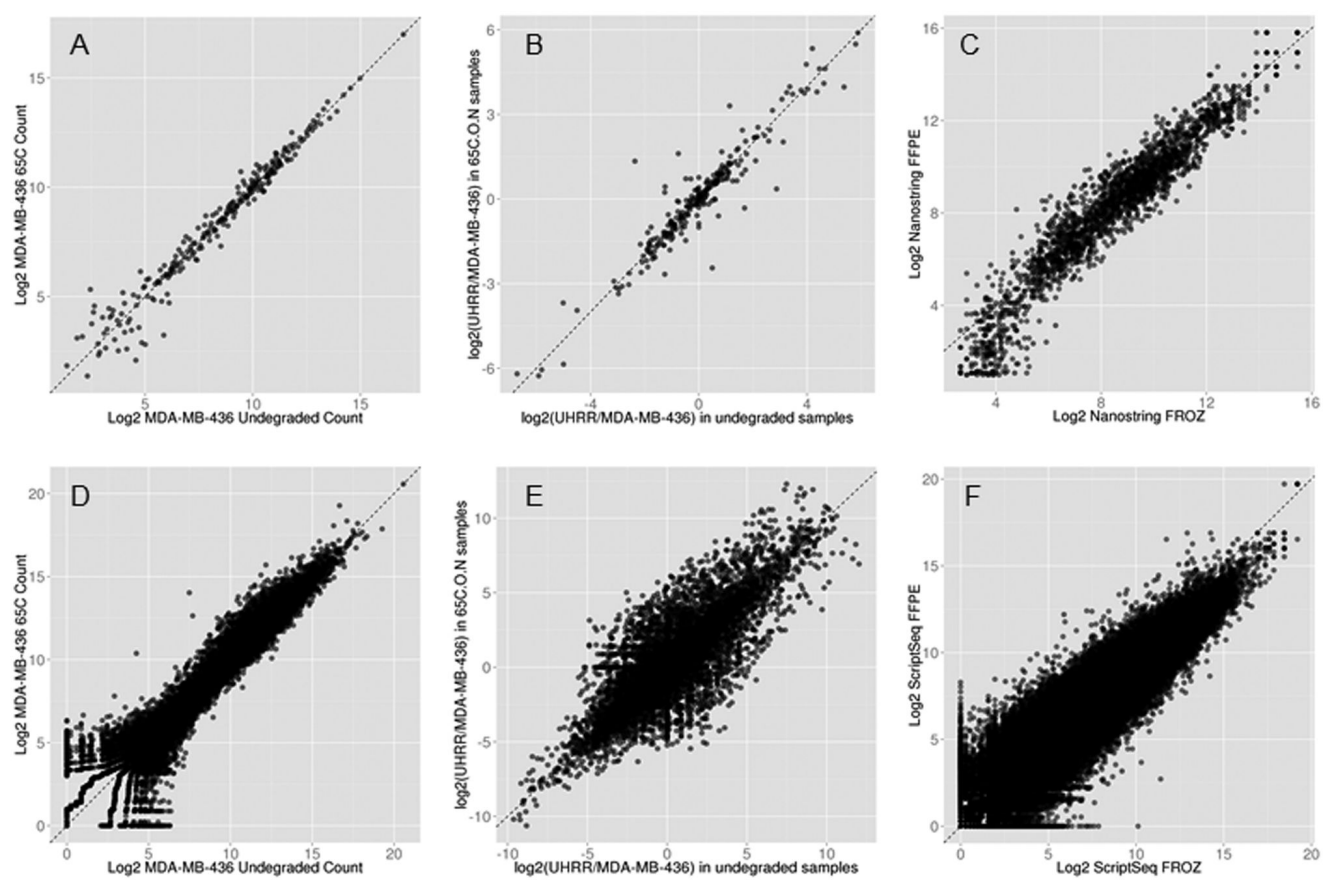
Gene Expression correlations with Nanostring, ScriptSeq And TruSeq platforms. Nanostring correlation for (A) undegraded RNA against the same manually degraded sample (RIN=2.0); (B) log_2_ fold change between two high quality RNAs and the same two samples when manually degraded (RIN 2.0); (C) nine matched fresh-frozen and FFPE pairs. ScriptSeq correlation for (D) undegraded RNA against the same manually degraded sample (RIN=2.0); (E) log_2_ fold change between two high quality RNAs and the same two samples when manually degraded (RIN 2.0); (F) nine matched fresh-frozen and FFPE pairs.

Raw gene counts for 226 genes quantified by NanoString were log_2_-transformed, and normalized by quantile normalization. For each high quality undegraded RNA isolated from cell lines (MDA-MB-436 and UHRR), we compared these values against the same sample that had been manually degraded to different extents with either heat or shearing. Excellent correlation was observed for both samples regardless of the state of degradation. Medium degradation (RIN scores 4.8-6.2) yielded Pearson correlations ranging 0.996 to 0.999. High degradation (RIN scores 1.2-2.2) yielded Pearson correlations ranging 0.993-0.998 (Figure 1A). 

We also performed correlation of the log_2_ fold changes between MDA-MB-436 and UHRR in their undegraded state verses the log_2_ fold changes in each state of degradation to test if the differences in gene expression between the two samples would also be detected when the samples were degraded. Again, high correlation was observed with Pearson correlation r= 0.943-0.960 and r=0.925-0.949 for medium and high states of degradation respectively (illustrated in Figure 1B).

**Figure 2:**
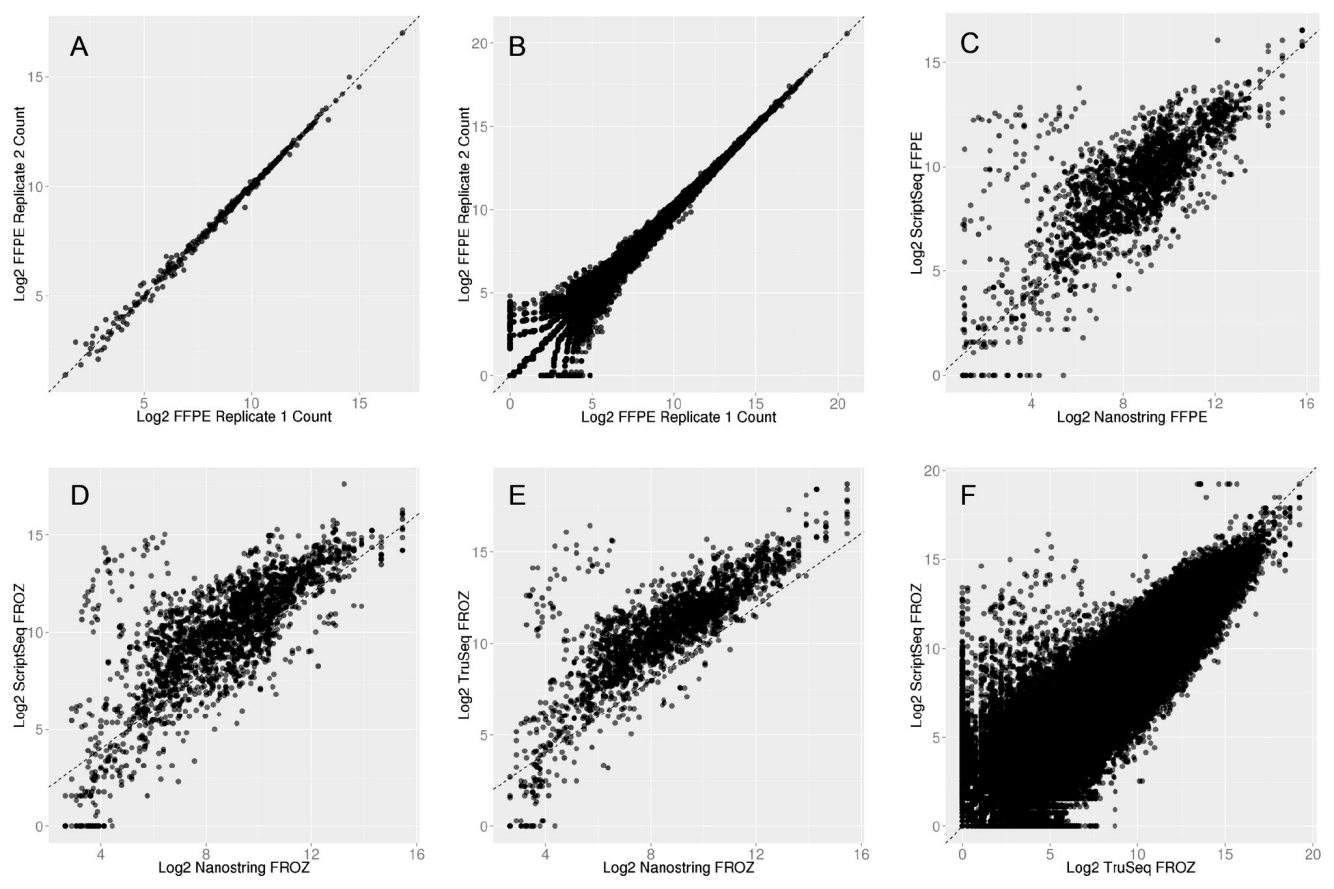
Technical Replicates and Cross-Platform Correlations. Log_2_ gene expression for FFPE technical replicate with (A) NanoString platform and (B) RiboZeroGold ScriptSeq; (C) Gene expression correlation of nine FFPE samples across nanoString and RiboZeroGold ScriptSeq protocols. Gene expression correlation of nine fresh-frozen (FROZ) RNA samples across (D) nanoString and ScriptSeq, (E) nanoString and TruSeq and (F) RiboZeroGold ScriptSeq and TruSeq.

To test technical reproducibility on the NanoString we performed two technical replicates of the same FFPE sample. Pearson correlation for technical replicates was 0.996 (Figure 2A).

In the second test set of nine matched pairs of fresh-frozen and FFPE RNAs, normalized log_2_ values for all 226 genes were compared by Pearson correlation and log rank correlation (Spearman). The NanoString platform showed excellent correlation between all pairs (Table 3 and Figure 1C) of fresh-frozen and FFPE RNA, Pearson correlation ranging 0.728-0.973 (mean 0.874, p<2x10^-16^).

**Table 3:** Pearson correlation between fresh frozen and FFPE RNA pairs using nanoString and ScriptSeq protocols.

	NanoString FROZ vs FFPE	ScriptSeq FROZ vs FFPE
BRB123	0.926 (0.950)	0.790 (0.943)
BRB144	0.955 (0.965)	0.807 (0.957)
BRB147	0.728 (0.917)	0.802 (0.945)
BRB157	0.807 (0.930)	0.793 (0.932)
BRB212	0.972 (0.962)	0.830 (0.963)
BRB215	0.818 (0.976)	0.598 (0.961)
BRB248	0.942 (0.976)	0.830 (0.972)
BRB277	0.973 (0.970)	0.825 (0.971)
MCJBCR028	0.747 (0.941)	0.773 (0.933)
mean	0.874 (0.954)	0.783 (0.953)

Spearman correlation in parentheses

### Whole transcriptome gene expression: RiboZeroGold / ScriptSeq V2 libraries

In this set of experiments, we assessed a protocol for whole transcriptome sequencing of manually degraded and FFPE RNA samples. The RiboZeroGold / ScriptSeq protocol is designed to avoid 3’ bias due to fragmented or degraded material. We measured the gene body coverage by plotting read depth from 5’ to 3’ across all genes (Figure S3). Gene body profiles were similar regardless of the degree of degradation suggesting that 3’ bias due to sample degradation is not an issue with the RiboZeroGold/ScriptSeq protocol. We further tested this observation by correlation of gene expression for each manually degraded RNA against its undegraded self (an example of which is shown in Figure 1D). Average Pearson correlation for MDA-MB-436 for pairs of undegraded verses degraded RNA were 0.945 under medium degradation (RIN 4.8-6.2) and 0.922 when highly degraded (RIN 1.2-2.2). For UHRR, average Pearson correlation for medium and high degradation were 0.809 and 0.805, respectively, demonstrating that under this RNA-Seq protocol, gene expression can be reliably quantified in degraded RNA. 

We also performed correlation of the log_2_ fold changes between MDA-MB-436 and UHRR in their undegraded state verses the log_2_ fold changes in each state of degradation to test if the differences in gene expression between the two different samples would also be detected when the samples were degraded. Again, high correlation was observed with Pearson correlation r= 0.864-0.868 and r=0.826-0.862 for medium and high states of degradation respectively (Figure 1E).

We next tested reproducibility by performing a technical replicate for a single FFPE sample with the RiboZeroGold ScriptSeq protocol. Pearson correlation for FFPE technical replicates was extremely high, r=0.998 (Figure 2B). Next, we compared nine matched pairs of fresh-frozen and FFPE tumor for gene expression with the same protocol. Pearson correlation for gene expression between FFPE and fresh-frozen RNA ranged from 0.598-0.830 (mean 0.783, p<2x10^-16^), (Figure 1F, Table 3). 

### Cross-platform gene expression: NanoString, ScriptSeq V2 and TruSeq V2

In this experiment, we performed comparisons of the same samples across different platforms. Additionally for this experiment we included data generated by polyA pull-down (TruSeq V2) for the nine RNA samples isolated from fresh-frozen tumor only. Correlation coefficients for all comparisons are shown in Table 4. Pearson correlation of whole transcriptome gene expression values for the same high quality (fresh-frozen) RNA samples between ScriptSeq and TruSeq ranged 0.579-0.821 (mean 0.680), Figure 2F. 

**Table 4:** Pearson cross-platform correlation.

	NanoString FFPE vs ScriptSeq FFPE	NanoString FROZ vs ScriptSeq FROZ	NanoString FROZ vs TruSeq FROZ	ScriptSeq FROZ vs TruSeq FROZ
BRB123	0.921 (0.771)	0.721 (0.740)	0.881 (0.829)	0.702 (0.931)
BRB144	0.924 (0.707)	0.746 (0.707)	0.710 (0.779)	0.579 (0.920)
BRB147	0.468 (0.727)	0.372 (0.734)	0.780 (0.822)	0.66 (0.920)
BRB157	0.869 (0.751)	0.805 (0.728)	0.880 (0.820)	0.682 (0.915)
BRB212	0.919 (0.711)	0.664 (0.714)	0.903 (0.811)	0.651 (0.929)
BRB215	0.744 (0.767)	0.634 (0.762)	0.610 (0.830)	0.8212 (0.942)
BRB248	0.889 (0.721)	0.679 (0.724)	0.922 (0.800)	0.686 (0.926)
BRB277	0.887 (0.717)	0.827 (0.730)	0.870 (0.794)	0.655 (0.934)
MCJBCR028	0.923 (0.731)	0.525 (0.743)	0.558 (0.801)	0.686 (0.942)
mean	0.838 (0.734)	0.664 (0.731)	0.790 (0.809)	0.680 (0.929)

Spearman correlation in parentheses

Both ScriptSeq and TruSeq protocols behaved similarly when compared against 226 genes analyzed on the NanoString platform, mean correlations r= 0.664 and 0.790, respectively (Figures 2D and 2E). The highest correlation was observed between FFPE samples comparing NanoString and ScriptSeq, Pearson correlation ranging 0.468-0.923 (mean 0.839), Figure 2C. When comparing both RNA-Seq protocols against NanoString, we observed a small cluster of outliers in both Figure 2D and 2E from the same five genes in all samples (*AKT1*, *ATM*, *GNAS*, *NPM1* and *WEE1*). We assessed transcript length, GC content and GGGG motif’s within these five transcripts to identify possible factors that might account for the outliers, but no common factor was observed; and the values for each of these parameters were close to the median value observed across all 226 genes in the NanoString panel (data not shown).

### RNA-Seq protocol comparison

Correlative studies of gene expression with FFPE material have previously been demonstrated with a number of different platforms such as NanoString, microarrays and quantitative PCR [5,6,13]. In addition to quantifying gene expression at the level of the transcriptome, RNA sequencing carries the additional benefit of expressed SNV detection and detection of fusion transcripts. We used the RNA-Seq datasets generated for TruSeq and RiboZeroGold ScriptSeq libraries from nine fresh-frozen tumor samples and RiboZeroGold ScriptSeq libraries generated from nine matched FFPE tumors to separate differences due to protocol and differences due to sample degradation, which allowed us to quantify the sensitivity of eSNV and fusion transcript detection in FFPE material.

### RNA-Seq mapping statistics

We compared sequencing statistics for the same nine fresh-frozen tumor samples using the TruSeq (polyA pull-down) and RiboZeroGold (rRNA depletion) ScriptSeq protocols and between matched fresh-frozen and FFPE samples for the RiboZeroGold ScriptSeq protocol. 

**Figure 3:**
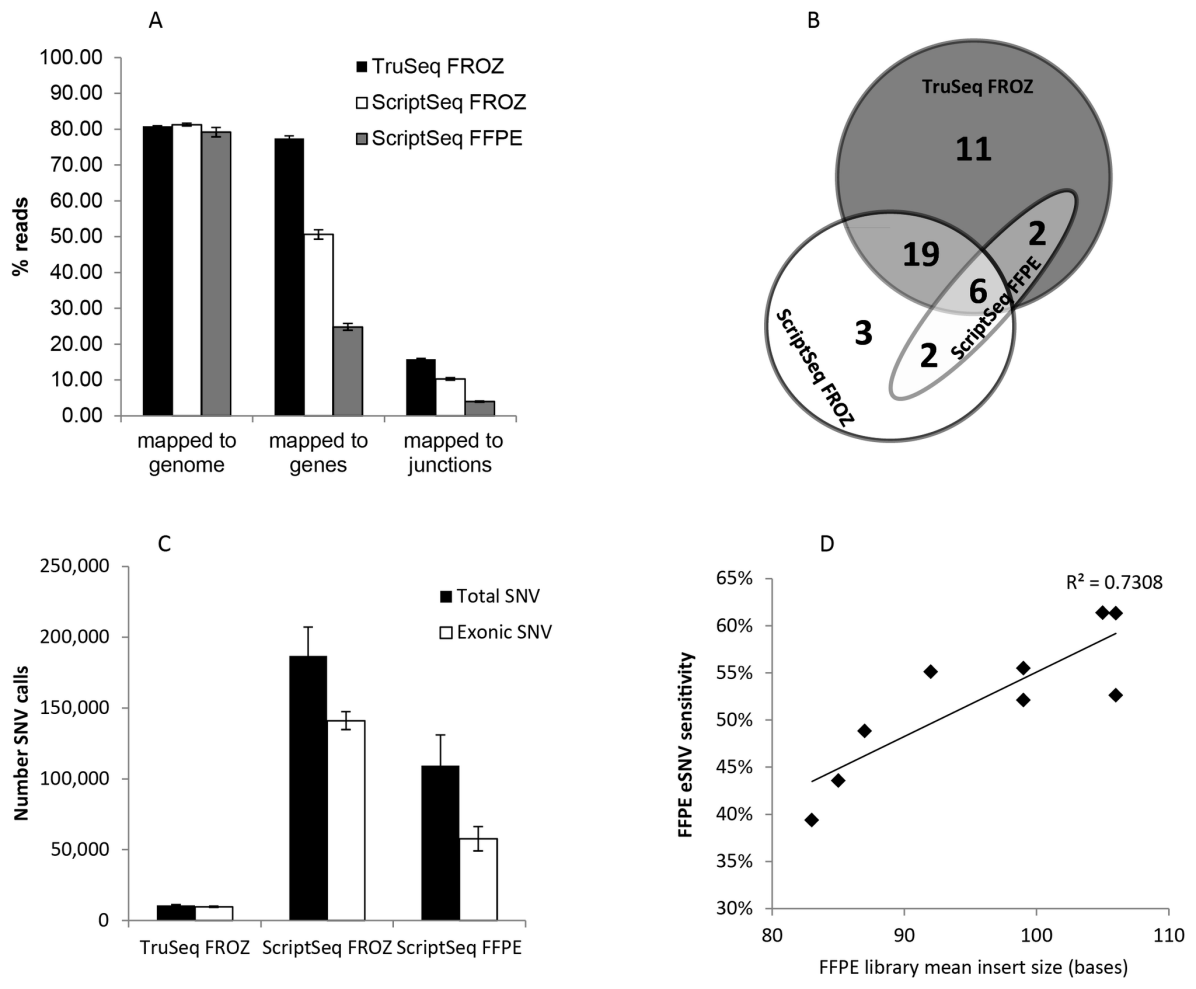
RNA-Seq Mapped Reads, Fusion and eSNV statistics By Protocol. (A) % reads mapped to genome, genes and exon junctions, B) Venn diagram of fusion transcript detection, (C) Number SNV calls, (D) Correlation of FFPE library insert size with sensitivity for single nucleotide variant detection.

This resulted in two observations illustrated in Figure 3A. We observed a difference between the TruSeq and ScriptSeq protocols. The number of reads mapping to the genome was almost identical for fresh-frozen samples regardless of protocol, mean 80.8% (SD ±0.48) for TruSeq and 81.3% (SD ±1.12) for ScriptSeq. However, the percentage of reads mapped within genes and to exon junctions was higher for the TruSeq protocol. The TruSeq mean percentage of reads mapped to genes and exon junctions was 77.4%, SD ± 0.74 and 15.8% SD ±0.20, respectively, compared to 50.6%, SD ±1.32 and 10.29%, SD 0.36, respectively for ScriptSeq, suggesting the TruSeq protocol yields a higher percentage of reads mapping to coding genes and ScriptSeq yields a greater proportion of reads mapping to intronic and intergenic regions. 

We also observed a difference between fresh-frozen and FFPE material that was not due to protocol. The total number of reads mapped to the genome for nine FFPE samples was 79.2%, similar to that of fresh-frozen material with either protocol. When comparing the same RiboZeroGold ScriptSeq protocol across matched fresh-frozen and FFPE samples, we observed a significantly lower percentage of reads mapping to genes (fresh-frozen = 50.6%, SD ± 3.95 and FFPE = 24.8%, SD ±2.8) and to exon junctions (fresh-frozen = 10.29%, SD ±1.09 and FFPE = 3.98%, SD ±0.58), in FFPE samples, demonstrating a marked loss of exonic sequence in FFPE material.

### RNA-Seq protocol specific differential gene expression

Comparison of TruSeq verses RiboZeroGold ScriptSeq libraries from the same fresh-frozen tumor allowed us to examine differences in gene expression that were protocol specific. Firstly, we identified outliers by assessing log_2_ fold change between the same nine samples. This identified 116 genes (Table S2) that were detected with both protocols but were outliers in all nine samples. 107/116 (92.2%) of outliers were expressed more highly (>1.5*IQR) in the RiboZeroGold ScriptSeq libraries than in the TruSeq libraries. 23.4% of outliers with expression in ScriptSeq libraries encoded histone H1 cluster genes known to lack polyA tails and a further 26.2% encoded small nucleolar RNA and small Cajal body-specific RNAs, which are non-coding, demonstrating a greater diversity of genes observed in the RiboZeroGold ScriptSeq libraries. 

### Long Intergenic Non-Coding RNAs (lincRNAs)

We also aligned RNA-Seq data from both TruSeq and RiboZeroGold ScriptSeq libraries against 5,749 lincRNAs from the Havana group (http://www.sanger.ac.uk/research/projects/vertebrategenome/havana/) and performed correlation analyses of the nine fresh-frozen and FFPE pairs, (Table 5). 

**Table 5:** lincRNA: mapped reads and Pearson correlation.

	% reads mapped to lincRNA			lincRNA correlation of expression	
	ScriptSeq FFPE library	ScriptSeq FROZ library	TruSeq FROZ library	ScriptSeq FROZ vs ScriptSeq FFPE	TruSeq FROZ vs ScriptSeq FROZ
BRB123	5.74%	3.68%	1.66%	0.997 (0.858)	0.465 (0.77)
BRB144	4.93%	3.80%	1.30%	0.99 (0.881)	0.361 (0.716)
BRB147	6.32%	3.23%	1.10%	0.98 (0.835)	0.376 (0.677)
BRB157	5.80%	3.72%	1.14%	0.969 (0.818)	0.36 (0.641)
BRB212	4.70%	3.44%	1.26%	0.996 (0.896)	0.366 (0.74)
BRB215	4.44%	2.63%	1.39%	0.989 (0.855)	0.382 (0.716)
BRB248	5.94%	4.89%	1.85%	0.992 (0.879)	0.355 (0.737)
BRB277	5.55%	3.49%	1.42%	0.995 (0.874)	0.486 (0.717)
MCJBCR028	5.84%	3.37%	1.67%	0.982 (0.849)	0.477 (0.807)
mean	5.47%	3.58%	1.42%	0.988 (0.861)	0.403 (0.725)

Spearman correlation in parentheses

The percentage of reads mapping to lincRNA from ScriptSeq FFPE libraries ranged from 4.44-6.32% (mean 5.47%), better than that achieved in the matched fresh-frozen libraries using the same ScriptSeq protocol (range 2.63-4.89%, mean 3.58%) and markedly better than that achieved using the TruSeq protocol (range 1.10-1.85%, mean 1.42%). This observation was mirrored when comparing the percent of total lincRNAs present in each library (ScriptSeq FFPE mean % of total lincRNA = 57.57%, compared with 55.80% and 42.73% for matched fresh-frozen libraries with ScriptSeq and TruSeq protocols respectively).

We observed excellent correlation of lincRNA expression between the nine matched FFPE and fresh-frozen pairs under the RiboZeroGold ScriptSeq protocol (Pearson correlation ranging 0.969-0.997, mean correlation r=0.989). Correlation between libraries prepared from fresh-frozen material with the ScriptSeq and TruSeq protocols was relatively poor, (Pearson correlation ranging 0.355-0.477, mean correlation 0.403). We further examined the lincRNA outliers (>1.5*IQR) between ScriptSeq and TruSeq protocols (Table S3). 251 lincRNAs were outliers in at least one pair, of which 72 were more highly expressed in the TruSeq libraries and 179 were more highly expressed in the ScriptSeq libraries. In those outliers with higher expression in ScriptSeq libraries, transcript size was markedly longer (mean transcript length 136kb, median transcript length 60kb) compared to outliers expressed more highly in the TruSeq libraries (mean transcript length 14.4kb, median transcript length 2.30kb). 

### Fusion genes

We applied the snowshoes-FTD, fusion transcript detection [12] to nine fresh-frozen (TruSeq and RiboZeroGold ScriptSeq protocols) and nine matched FFPE tumor RNAs prepared with the RiboZeroGold ScriptSeq protocol only. The same parameters were used for both fresh-frozen and FFPE RNA sequence. Details of fusion transcripts identified by at least two protocols are shown in Table S4. 

Overlap between protocols and samples are shown by Venn diagram in Figure 3B. The TruSeq protocol identified 38 fusions, whereas the ScriptSeq protocol identified 30. To determine the sensitivity of fusion detection in FFPE material, we defined high confidence fusion transcripts as those that overlapped between TruSeq and ScriptSeq libraries in the same RNA samples isolated from fresh-frozen tumor. We defined sensitivity in FFPE libraries as the fraction of fusion transcripts detected as a proportion of the high confidence calls observed in the matched fresh-frozen RNA libraries. 25 fusion transcripts were detected by both ScriptSeq and TruSeq protocols in the same fresh-frozen samples, of which 6/25 (24%) were detected in matched FFPE ScriptSeq libraries. In addition, two fusion transcripts (ARID4B>LRRN2 and ERBB2>TOM1L1) were detected in TruSeq libraries from frozen material and ScriptSeq FFPE libraries but not in ScriptSeq frozen libraries.

We next asked the question, could the sensitivity to detect fusion transcripts in FFPE libraries be improved by increased depth of coverage? Of the 19 fusions identified in both TruSeq and ScriptSeq libraries from fresh-frozen material, only 2/19 were detected in FFPE material when using all available reads, in this case ~159 million (~ three-fold increase). We also note that for exactly the same FFPE library (BRB-277), 4/19 fusion transcripts present in the matched fresh-frozen libraries prepared with TruSeq and ScriptSeq were not identified in the FFPE library, despite increasing read depth to 159 million reads. These data suggest that an increase in read depth, at least up to ~3-fold, does not compensate for FFPE specific exonic loss.

### Expressed single nucleotide variants (eSNVs)

The library preparation methods used in this study employ different techniques for mRNA enrichment and cDNA synthesis, as demonstrated by a decrease of ~27% of reads mapped to RefSeq genes and exon junctions between TruSeq and ScriptSeq protocols in the same high quality RNA samples (Figure 3A) and the significantly higher number of SNV calls in all ScriptSeq libraries (Figure 3C). 

As a measure of data quality, we calculated Transition / transversion (Ti/Tv) ratios for known and novel SNV’s and mean quality scores for the alternate allele. Ti/TV ratios have been previously reported as ~3.0 for SNVs inside of exons, and ~2.0 elsewhere in the genome, [14]. Ti/Tv ratios in both fresh-frozen and FFPE datasets lie within this range, with some differences between the ScriptSeq and TruSeq protocols, and some differences between fresh-frozen and FFPE samples. For known SNV, the fresh-frozen TruSeq libraries showed the highest Ti/Tv ratio (fresh-frozen TruSeq, mean Ti/Tv 2.44, range 2.4-2.51; fresh-frozen ScriptSeq, mean Ti/Tv 2.15, range 2.06-2.27; FFPE ScriptSeq, mean Ti/Tv 2.21, range 2.17-2.25). For novel SNV, the highest Ti/Tv ratio was observed in the FFPE samples (fresh-frozen TruSeq, mean Ti/Tv 1.91, range 1.51-2.39; fresh-frozen ScriptSeq, mean Ti/Tv 1.81, range 1.52-2.39; FFPE ScriptSeq Ti/Tv ratio 2.23, range 2.08-2.39). All Ti/Tv ratios are given in Table S5.

The mean quality score for alternate alleles was similar across all samples sets and protocols, ranging 83.3-86.9 in the TruSeq fresh-frozen samples, 87.9-89.5 in the ScriptSeq fresh-frozen samples and 85.7-86.6 in the ScriptSeq FFPE samples. Mean and median quality scores for alternate alleles and coverage for alternate alleles are shown in detail in Table S5.

To assess the sensitivity of SNV detection in our FFPE samples, we defined high confidence SNV calls as those that overlapped between TruSeq and ScriptSeq libraries in the same RNA samples isolated from fresh-frozen tumor. We defined sensitivity of SNV detection in FFPE libraries as the fraction of SNVs detected as a proportion of the high confidence SNV calls observed in the matched fresh-frozen RNA libraries. Sensitivity to detect SNV in FFPE material ranged from 39-61% and was correlated with library insert size, R^2^ = 0.73 (Figure 3D) with smaller insert size correlating with reduced sensitivity to detect SNV’s. 

Comparison of fresh-frozen and FFPE libraries made with the same RiboZeroGold ScriptSeq protocol, (Figure 3C) also identified a higher proportion of non-exonic SNV detected in FFPE libraries, (mean 53.4%, SD +/- 5.0% in FFPE and mean 76.2% SD +/- 6.6% in fresh-frozen libraries), similar to the difference observed in percentage of reads mapping to non-genic regions (Figure 3A).

## Discussion

Discovering the unique genomic architecture within tumors will be essential to the goal of personalized medicine for breast cancer. Next generation sequencing approaches applied to large clinical samples with detailed phenotypic data are potentially invaluable but sample preservation by formalin-fixation has hindered this application. In this study we assessed the feasibility of RNA sequencing of formalin-fixed material for gene expression including lincRNA expression, discovery of single nucleotide variants and discovery of fusion transcripts. 

RNA sequencing of FFPE material is not possible with methods that require mRNA enrichment by polyA pull-down due to sample degradation. Alternative protocols and platforms are available, but differentiation between artifact due to FFPE and artifact due to different protocols and platforms is equivocal. In this study we break down these differences. We used both a hybridization based platform (NanoString nCounter) and a rRNA depletion protocol for transcriptome sequencing (RiboZeroGold ScriptSeq library preparation with Illumina sequencing) in a set of manually degraded RNAs and a set of nine matched fresh-frozen and FFPE pairs. Correlative analyses of matched fresh-frozen and FFPE pairs on the same platform and for the same samples (both fresh-frozen and FFPE) across platforms demonstrates 1) gene expression data from RNA isolated from FFPE breast tumor is viable; 2) Both the NanoString platform and RiboZeroGold ScriptSeq whole transcriptome sequencing protocols generate reliable gene expression data for degraded and FFPE RNA. 

Our initial study design systematically assessed gene expression as a function of RNA integrity, by correlation of a set of manually degraded RNA samples against undegraded, high quality aliquots of the same sample. Both platforms performed well with no systematic difference between degraded and undegraded states. RNA expression study designs depend on accurate estimation of fold change in expression between different samples. By using two different samples, one from a breast cancer cell line and a universal human reference RNA (a pool of ten different cell lines), we were also able to correlate log_2_ fold change between samples in their undegraded and degraded states, further demonstrating the robustness of both the NanoString nCounter platform and the RiboZeroGold ScriptSeq protocol. 

Correlative analyses with manually degraded samples were encouraging for both platforms. However, it is unknown if these artificial changes are representative of the biological changes that we seek in degraded material. Certainly, they do not account for the cross-linking caused by formalin fixation. We next moved to assess three critical issues for gene expression studies in FFPE samples. Firstly, we used technical replicates of RNA isolated from FFPE to assess reproducibility with the NanoString and ScriptSeq protocols, which yielded Pearson correlation >0.99 for both platforms. Secondly, for study designs using a discovery sample from FFPE material, we need to know that the genes that appear to be associated with disease outcome, are not systematic artifacts of FFPE material. This issue was assessed for both platforms by correlation of gene expression in matched pairs of fresh-frozen and FFPE RNA. In agreement with Reis et al (2011), we observed a high degree of correlation between fresh-frozen and FFPE pairs with the NanoString platform, Pearson r=0.874. Using the RiboZeroGold/ScriptSeq protocol, fresh-frozen and FFPE pairs showed slightly lower correlation, (Pearson r=0.783), although significantly better than that reported with quantitative PCR, (Pearson r=0.53), [6]. 

The third gene expression issue we addressed relates to orthogonal platform validation. Genome-wide studies in discovery samples will generate a high number of disease or disease associated genes, requiring validation and further replication samples. For this reason, it is important to identify genome-wide and replication platforms which achieve high correlation for FFPE material. The two protocols we assessed in this study are very different. The RNA-Seq protocol is based on rRNA depletion and PCR amplification of the cDNA, whereas the NanoString protocol is based on hybridization of selected probes to total RNA, with no cDNA synthesis and no PCR amplification. Despite these differences, these platforms showed excellent correlation for nine FFPE samples, Pearson r=0.838. We believe this combination, ScriptSeq then NanoString is ideally suited for discovery and analytical validation, respectively.

We next moved to assess the performance of a rRNA depletion protocol for whole transcriptome sequencing of FFPE RNA. By generation of libraries from the same nine RNAs isolated from fresh-frozen tumor, with both TruSeq and ScriptSeq complete kits, we were able to separate those characteristics due to protocol from those due to FFPE. Comparison of sequencing statistics between RNA-Seq protocols revealed a marked (~50%) decrease in the percentage of reads mapping to RefSeq genes and exon junctions in FFPE material, that was not observed in the matched frozen and manually degraded libraries prepared with the same RiboZeroGold ScriptSeq protocol. The exonic loss from RiboZeroGold ScriptSeq FFPE libraries was also reflected in the lower number of fusion transcripts detected, although a significant number (22.2% of total detected fusions) were still identified. 

When comparing the same fresh-frozen samples with TruSeq and RiboZeroGold ScriptSeq protocols, the TruSeq libraries generated a higher percentage of reads mapping to genes, exon junctions and hence a higher number of fusion transcripts. 86.7% of total fusion transcripts identified were present in the TruSeq libraries compared to 68.9% in the ScriptSeq libraries for the same high quality RNA sample. To make these comparisons, we extracted ~56 million reads from each sample (the number of reads observed across all samples ranged from 56 million to 361 million), such that the starting number of total reads was the same. Application of our fusion pipeline to the fully available reads for each sample suggest that an increase in read depth, at least up to ~3-fold would not compensate for these differences.

However, on the other side of the coin, the RiboZeroGold ScriptSeq protocol is suitable for low starting amounts of FFPE material and also generated a higher proportion of reads mapping to non-coding genes and genes lacking a polyA tail, an aspect which may prove an important characteristic of the breast cancer genome. This was demonstrated both in our own study where we observed superb correlation of lincRNA expression between matched fresh-frozen and FFPE pairs (mean Pearson r = 0.988) and a higher proportion of reads mapping to lincRNAs with the RiboZeroGold ScriptSeq protocol, and independently by Sinicropi et al, [9]. Sinicropi used a novel ribosomal RNA depletion method in combination with the ScriptSeq protocol to prepare libraries from FFPE tumor material of 136 breast cancer patients. The study identified 1,698 transcripts mapping to introns that were associated with breast cancer recurrence. 

Finally, we assessed the quality and sensitivity of SNV detection in our FFPE libraries compared to matched fresh-frozen pairs, and accounting for difference between TruSeq and ScriptSeq protocols. Transition:transversion (Ti/Tv) ratios of the RiboZeroGold ScriptSeq FFPE libraries were within the range reported from DNA sequencing studies [14,15], and highly similar to their matched RiboZeroGold ScriptSeq counterparts for known SNVs, 2.21 and 2.15 respectively. However, for novel SNVs, the Ti/Tv ratio was slightly higher in FFPE than fresh-frozen material, 2.23 and 1.81 respectively, likely a result of formalin-fixation. 

FFPE samples detected on average 52.2% of SNVs that were detected with high confidence in matched fresh-frozen samples. Lower sensitivity correlated with smaller library insert size, r^2^ = 0.73, a parameter that could be used to predict performance of SNV detection specifically for each FFPE sample. Performance of gene expression from FFPE material has previously been correlated with time of fixation rather than age of sample [2], although not with RNA-Seq or SNV detection and in reality, for many available FFPE samples, fixation time will be unknown. If the sensitivity can be predicted from parameters such as library insert size, this will enable power calculations on required sample size to identify genes with cancer associated SNV.

In conclusion, we observed a high correlation of gene expression between fresh-frozen and FFPE RNA with both hybridization based and next generation sequencing platforms. The high degree of cross-platform correlation suggests the NanoString nCounter^TM^ will provide an excellent platform for analytical validation of gene expression generated from FFPE whole transcriptome datasets. Our data suggest that RiboZeroGold ScriptSeq libraries are also suitable for eSNV and fusion transcript detection with some reduction in sensitivity, the proportion of which may be estimated by library insert size. Library preparation protocols that do not rely on polyA pull-down for mRNA enrichment may allow detection of non-coding genes and genes that lack polyA tails that may be relevant to cancer genomics. 

## Supporting Information

Figure S1 **A. Agilent profiles: total RNA from cell lines, MDA-MB-436 and UHRR in undegraded and manually degraded forms.** B. Agilent profiles: total RNA from nine FFPE samples and nine matched fresh-frozen tissue samples.
(DOCX)

Figure S2 **A. Nanostring log_2_ gene count distributions of fresh-frozen (FROZ) and FFPE samples before and after quantile normalization.** **B**. RiboZeroGold ScriptSeq log_2_ gene count distributions of fresh-frozen (FROZ) and FFPE samples before and after quantile normalization.
(DOCX)

Figure S3 **Gene body plots 5’ to 3’ for TruSeq and RiboZeroGold/ ScriptSeq libraries from fresh-frozen breast tumor, and RiboZeroGold/ScriptSeq libraries from matched FFPE tumor.**
(DOCX)

Table S1 **Clinical data for each tumor.**
(XLSX)

Table S2 **Comparison of log_2_ fold change between TruSeq and RiboZeroGold ScriptSeq libraries from RNA isolated from fresh-frozen tumor identified 116 genes that were outliers in all nine patients.**
(XLSX)

Table S3 Outliers in lincRNA genes between ScriptSeq and TruSeq libraries constructed from RNA isolated from fresh-frozen tumor (outliers were defined determined as gene expression >1.5*IQR).
(XLSX)

Table S4 **Fusion transcripts identified by at least two RNASeq protocols.**
(XLSX)

Table S5 **Transition/Transversion ratios, quality scores and coverage for single nucleotide variants in each library.**
(XLSX)
