# Supplementary material for: Gene Expression, Single Nucleotide Variant and Fusion Transcript Discovery in Archival Material from Breast Tumors
Source: PLoS One. 2013 Nov 22;8(11):e81925. doi: 10.1371/journal.pone.0081925 (PMC3838386; doi:10.1371/journal.pone.0081925)

**Figure S1A.** Agilent profiles: total RNA from cell lines, MDA-MB-436 and UHRR in undegraded and manually degraded forms.


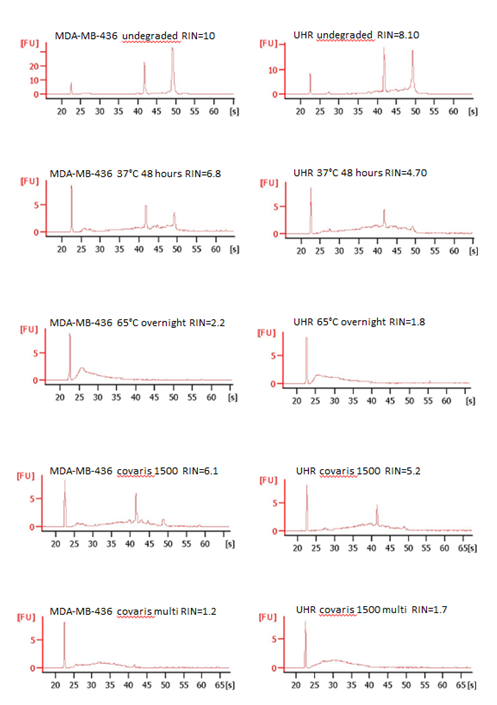


**Figure S1B.** Agilent profiles: total RNA from nine FFPE samples and nine matched fresh-frozen tissue samples.


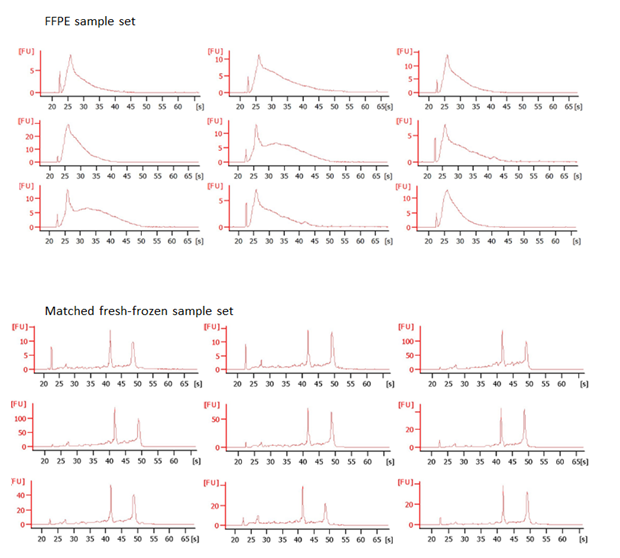

Supplement: Figure S1 — A. Agilent profiles: total RNA from cell lines, MDA-MB-436 and UHRR in undegraded and manually degraded forms. B. Agilent profiles: total RNA from nine FFPE samples and nine matched fresh-frozen tissue samples. (DOCX) [file pone.0081925.s001.docx]
