# Supplementary material for: Gene Expression, Single Nucleotide Variant and Fusion Transcript Discovery in Archival Material from Breast Tumors
Source: PLoS One. 2013 Nov 22;8(11):e81925. doi: 10.1371/journal.pone.0081925 (PMC3838386; doi:10.1371/journal.pone.0081925)

**Figure S2A.** Nanostring log2 gene count distributions of fresh-frozen (FROZ) and FFPE samples before and after quantile normalization.


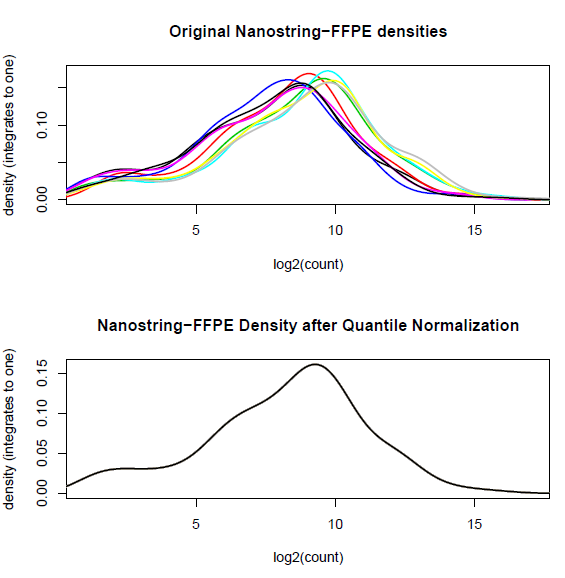

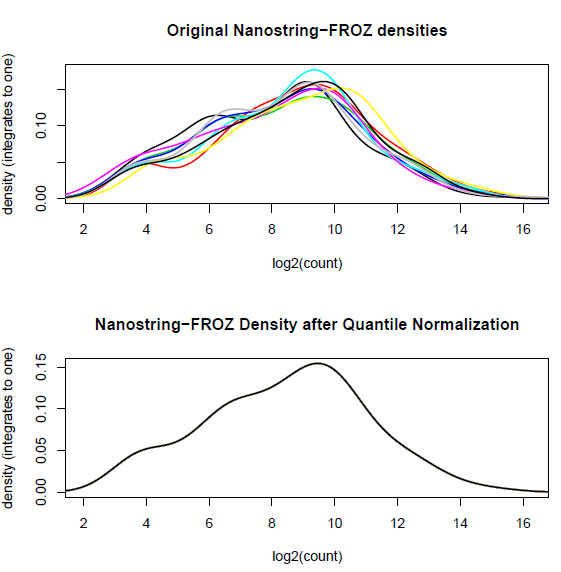


**Figure S2B.** RiboZeroGold ScriptSeq log2 gene count distributions of fresh-frozen (FROZ) and FFPE samples before and after quantile normalization.


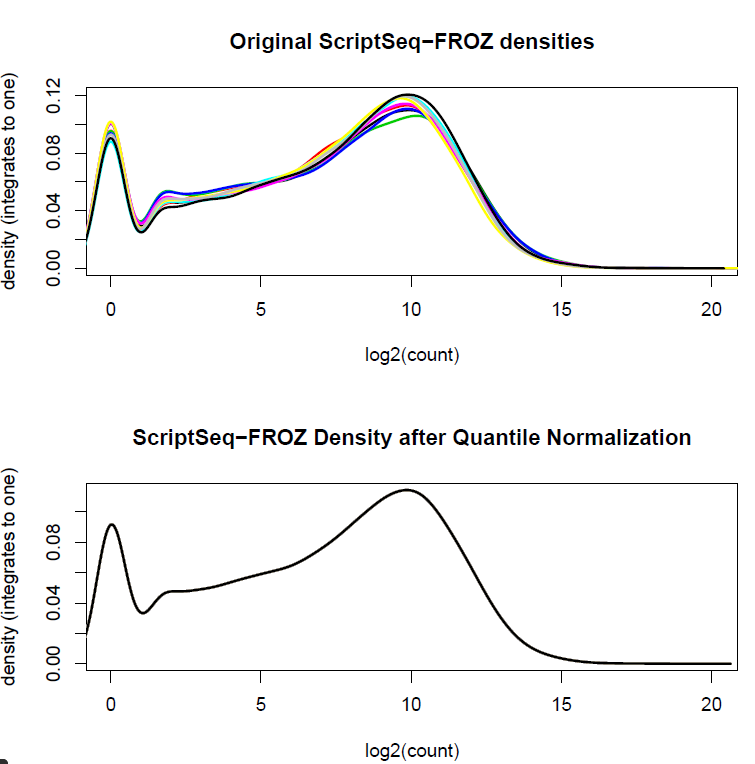

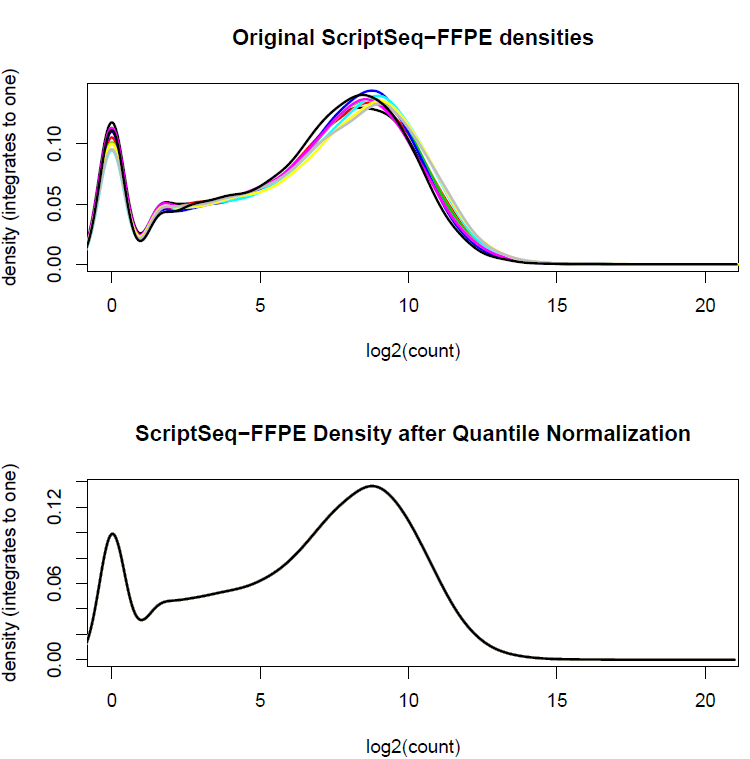

Supplement: Figure S2 — A. Nanostring log2 gene count distributions of fresh-frozen (FROZ) and FFPE samples before and after quantile normalization. B. RiboZeroGold ScriptSeq log2 gene count distributions of fresh-frozen (FROZ) and FFPE samples before and after quantile normalization. (DOCX) [file pone.0081925.s002.docx]
