# Supplementary material for: Gene Expression, Single Nucleotide Variant and Fusion Transcript Discovery in Archival Material from Breast Tumors
Source: PLoS One. 2013 Nov 22;8(11):e81925. doi: 10.1371/journal.pone.0081925 (PMC3838386; doi:10.1371/journal.pone.0081925)

**Figure S3.** Gene body plots 5’ to 3’ for TruSeq and RiboZeroGold/ ScriptSeq libraries from fresh-frozen breast tumor, and RiboZeroGold/ScriptSeq libraries from matched FFPE tumor.


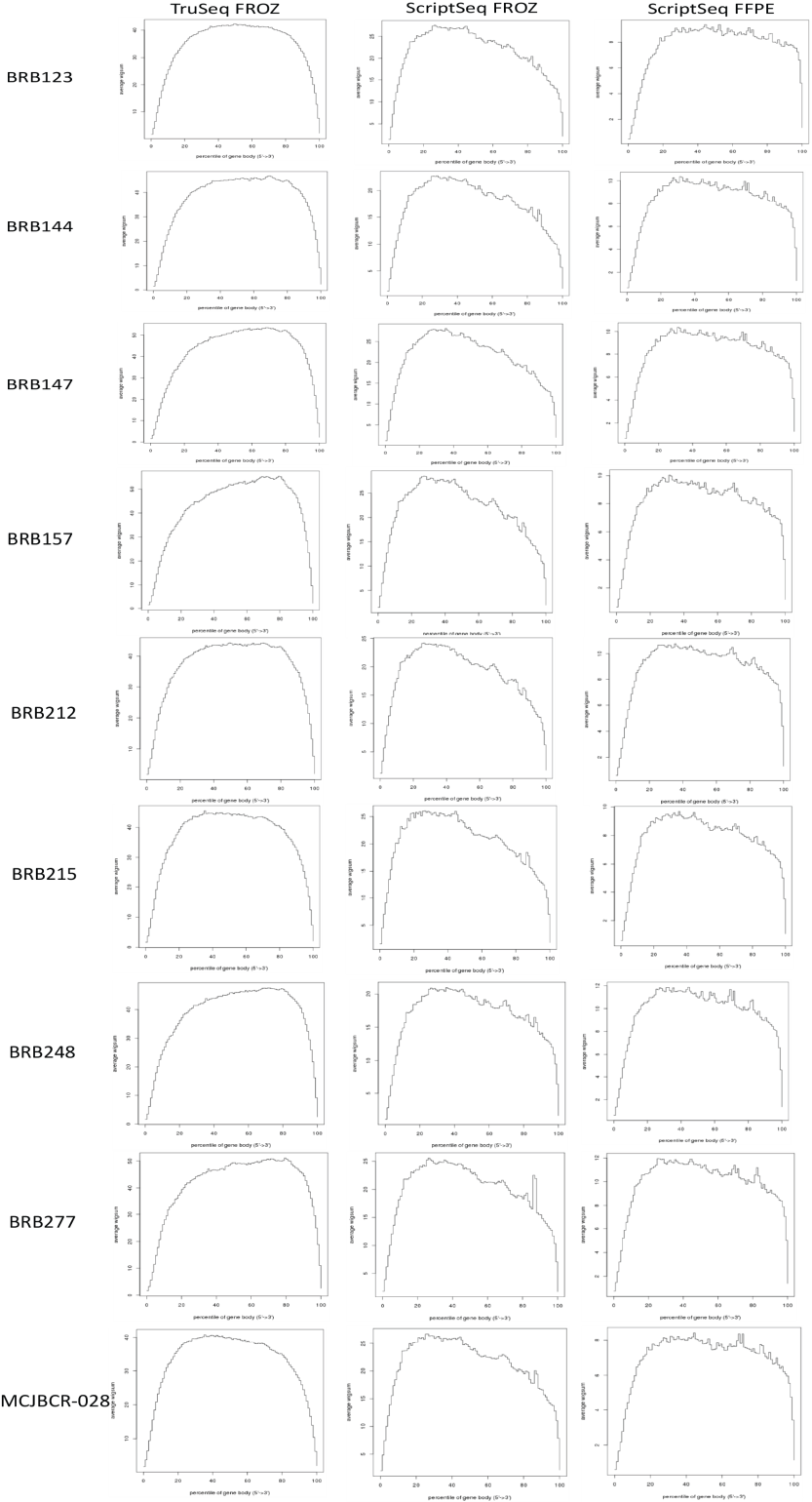

Supplement: Figure S3 — Gene body plots 5’ to 3’ for TruSeq and RiboZeroGold/ ScriptSeq libraries from fresh-frozen breast tumor, and RiboZeroGold/ScriptSeq libraries from matched FFPE tumor. (DOCX) [file pone.0081925.s003.docx]
